# Endophytic Strain *Bacillus subtilis* 26DCryChS Producing Cry1Ia Toxin from *Bacillus thuringiensis* Promotes Multifaceted Potato Defense against *Phytophthora infestans* (Mont.) de Bary and Pest *Leptinotarsa decemlineata* Say

**DOI:** 10.3390/plants9091115

**Published:** 2020-08-28

**Authors:** Antonina Sorokan, Galina Benkovskaya, Guzel Burkhanova, Darya Blagova, Igor Maksimov

**Affiliations:** Institute of Biochemistry and genetics of the Ufa Federal Research Centre of the Russian Academy of Sciences, 450054 Ufa, Russia; fourtyanns@googlemail.com (A.S.); bengal@yandex.ru (G.B.); guzel_mur@mail.ru (G.B.); blagova_darya@mail.ru (D.B.)

**Keywords:** *Bacillus subtilis*, *B. thuringiensis*, recombinant biopesticide, potato, *Leptinotarsa decemlineata*, *Phytophthora infestans*, priming, systemic resistance

## Abstract

Novel properties of a previously obtained *Bacillus subtilis* 26DCryChS strain are described. The *B. subtilis* 26DCryChS strain is able to produce Cry1Ia δ-endotoxin from *B. thuringiensis* B-5351 and to exist in internal plant tissues of potato plants in the same manner as the endophytic *B. subtilis* 26D source strain (487 ± 53 and 420 ± 63 CFU*10^3^/g, respectively). *B. subtilis* 26DCryChS, as much as the original *B. subtilis* 26D strain, inhibited mycelium growth of oomycete *Phytophthora infestans* (Mont.) de Bary and reduced late blight symptoms development on plants by 35% compared with non-treated ones, as well as showed insecticidal activity against *Leptinotarsa decemlineata*. Production of the fluorescent GFP protein in the *B. subtilis* 26D genome allowed visualizing the endophytes around damaged sites on beetle intestines. *Bacillus* strains under investigation induced systemic resistance to *P. infestans* and *L. decemlineata* through the activation of the transcription of PR genes in potato plants. Thus, the *B. subtilis* 26DCryChS strain was able to induce transcription of jasmonate-dependent genes and acquired the ability to promote transcription of a salicylate-dependent gene (PR1) in plants infected with the late blight agent and damaged by Colorado potato beetle larvae. The *B. subtilis* 26DCryChS strain could be put forward as a modern approach for biocontrol agents design.

## 1. Introduction

It is estimated that 35% of agricultural plants’ yield is lost due to biotic damage (insects, weeds, and pathogens) in the field around the world. Moreover, postharvest losses may constitute up to 15% of the total yields [1]. This has resulted in the complete dependence of crops on the use of chemical pesticides to reduce losses. Unfortunately, target organisms often develop resistance to globally used chemicals. At the moment, the most limiting productive capacity factor for potato is late blight caused by *Phytophthora infestans* (Mont.) de Bary infection. If left uncontrolled, late blight causes massive yield losses annually, especially under favorable conditions for *P. infestans* dispersion [2]. Since the last quarter of the century, different fungicides have been developed for the management of potato late blight disease, but the number of *P. infestans* isolates resistant to fungicides continuously increases. It is currently reported that most of the present-day *P. infestans* populations are fully resistant to the pesticide metalaxyl [3].

Capacity to develop insecticide resistance is the legendary feature of *Leptinotarsa decemlineata* Say [4]. The Colorado potato beetle is the most harmful pest of potato and its populations develop resistance to all major classes of insecticides, including δ-endotoxins produced by the most commonly used biocontrol agent *Bacillus thuringiensis* [5,6]. Multifaceted influences of management means on pathogens or pests are considered to slow down the development of their resistance to pesticides or biocontrol agents.

Plants are exposed to various harmful organisms simultaneously. Plant protection, in turn, demands an elevating amount of chemical pesticides upload that multiplies their negative influence on ecosystems and accelerates the development of pest resistance to chemicals. Thus, this suggests the necessity for the development of environmentally safe biocontrol agents that reveal combined biocidal impact on abundant pathogens and pests, and which will be capable to give rise to systemic resistance in host plants. Consequently, a beneficial and heterogeneous group of plant growth-promoting bacteria (PGPB), inhabiting the rhyzosphere, phyllosphere, and internal tissues of plants, is of great interest [7,8,9].

Endophytic PGPB have an advantage over rhizospheric and phyllospheric strains since inhabiting inside a plant’s tissues gives an ability to contact with the plant’s cells continually and to directly influence the plant host’s metabolism and, furthermore, penetrative pathogen or pest. This “hidden existence”, in addition, allows reducing the impact of the ambient environment on microorganisms. It has been revealed that certain PGPB strains not only stimulate both plant growth and resistance against pathogens and pests [10,11] but also show fungicidal [12], aphicidal [8,13], and insecticidal activities [14] as a consequence of synthesizing antibiotics and biosurfactants [15]. Among them, *Bacillus* strains are important resources for biocontrol agents development. *Bacillus thuringiensis* (Berliner) is a ubiquitous spore-forming bacterium which inhabits diverse dwelling places and has been considered as the most income-generating bioinsecticide during the last century [16]. The practical application of *B. thuringiensis* has been promoted as a result of pests’ resistance to chemical insecticides [16,17]. *B. thuringiensis* strains are vulnerable to UV light and flushing by rain precipitation [18,19]. Currently, the search for endophytic microorganisms producing insectotoxins, which inhabit the internal tissues of plants and are less influenced by environmental factors and more integrated in plant metabolism than rhyzo- and fillospheric PGPB, is of great interest.

At the moment, researchers are accepting that various pathogens and non-pathogenic microorganisms could serve as triggers for systemic acquired resistance (SAR) and induced systemic resistance (ISR). SAR is commonly challenged by biotrophic pathogens attack and involves salicylic acid (SA). It is generally accepted that non-pathogenic rhizospheric fungi or bacteria cause ISR when the plant interacts with them, and this process is carried out with the participation of jasmonic acid (JA)/ethylene (ET) and activates plants resistance against necrotrophic pathogens and leaf-chewing pests, such as *L. decemlineata* [11,20]. A lot of strains of *Bacillus* sp. are responsible for ISR development which does not require the SA-sensitive pathway but is dependent on JA, ethylene, and the regulatory gene NPR1. This mechanism is in accordance with the model for ISR elicited by *Pseudomonas* sp. At the same time, some *Bacillus* sp. can activate ISR through mechanisms independent of JA and NPR1 and dependent on SA [21]. Furthermore, ISR triggered by *Pseudomonas* sp. does not result in accumulation of transcripts of the defense gene PR1 in plants, whereas *Bacillus* sp.-elicited ISR can induce it [22]. Thus, the mechanisms of PGPB participation in ISR are not understood, in particular, ISR against oomycete *P. infestans*.

A number of bacterial determinants have recently been shown to be involved in ISR triggering. Thus, *Bacillus* sp. strains produce lipopeptides from different families: bacillomycin, surfactin, iturin, and fengycin [23]. It has been shown that some surfactin-producing *Bacillus* strains can elicit ISR to *Botrytis cinerea* in grape, strawberry, and tomato plants [24] and *Rhizoctonia solani* in lettuce [25]. Recently, aphicidal and insecticidal activity of surfactin produced by *B. subtilis* and surfactin-producing strain *B. subtilis* 26D has been shown [13,14,26].

Consequently, obtaining genetically engineered lines with an enhanced ensemble of beneficial properties is of essential interest due to their prospective value in pathogen and pest management [16]. Currently, some strains of *B. megaterium, P. fluorescens, E. coli, and B. subtilis* [27] have been genetically modified via insertion of Cry toxin genes. Importantly, insertion of the *Btcry218* gene of *Bacillus thuringiensis* into the genome of the *Burkholderia pyrrocinia* JK-SH007 strain promotes 80% mortality of silkworms *Bombyx mori* larvae 24 h after eating mulberry leaves immersed in *B. pyrrocinia* JK-SH007 suspension [28].

Previously, we obtained the novel recombinant *B. subtilis* 26DCryChS (RCAM04928, ARRIAM collection) strain that expresses the gene *Btcry1Ia* [29] encoding Cry1Ia δ-endotoxin originated from the *B. thuringiensis* B-5351 strain. It was reported that *B. subtilis* 26DCryChS can exist endophytically in wheat plants and synthesize lipopeptide surfactin as well as the source strain *B. subtilis* 26D and is capable of producing Cry1Ia δ-endotoxin from the donor of the *Btcry1Ia* gene *B. thuringiensis* B-5351 strain [13]. The *B. subtilis* 26DCryChS strain shows aphicidal activity against *Schizaphis graminum* as compared with both *B. thuringiensis* B-5351 and *B. subtilis* 26D strains and inhibited the growth of the wheat pathogen *Stagonospora nodorum* Berk. that causes Septoria blotch [13].

The aim of this work was to investigate the capacity of the recombinant *B. subtilis* 26DCryChS strain expressing the gene *Btcry1Ia* of Cry1Ia δ-endotoxin to protect potato plants (*Solanum tuberosum* L.) from oomycete *Phytophthora infestans* (Mont.) de Bary and pest *Leptinotarsa decemlineata* Say.

## 2. Results

### 2.1. Assay of Endophytic Properties of Bacillus Strains

The number of *B. subtilis* 26DCryChS colony forming units (CFU) in interior tissues of potato plants after surface sterilization amounts to approximately 500,000 per g of plant wet mass, no less than the amount of *B. subtilis* 26D alive cells (Table 1). The content of *B. thuringiensis* B-5351 cells in potato plants was substantially lower than the *B. subtilis* strains under investigation.

### 2.2. Capacity of Bacillus Strains to Protect Potato Plants against Oomycete P. infestans

**Figure 1:**
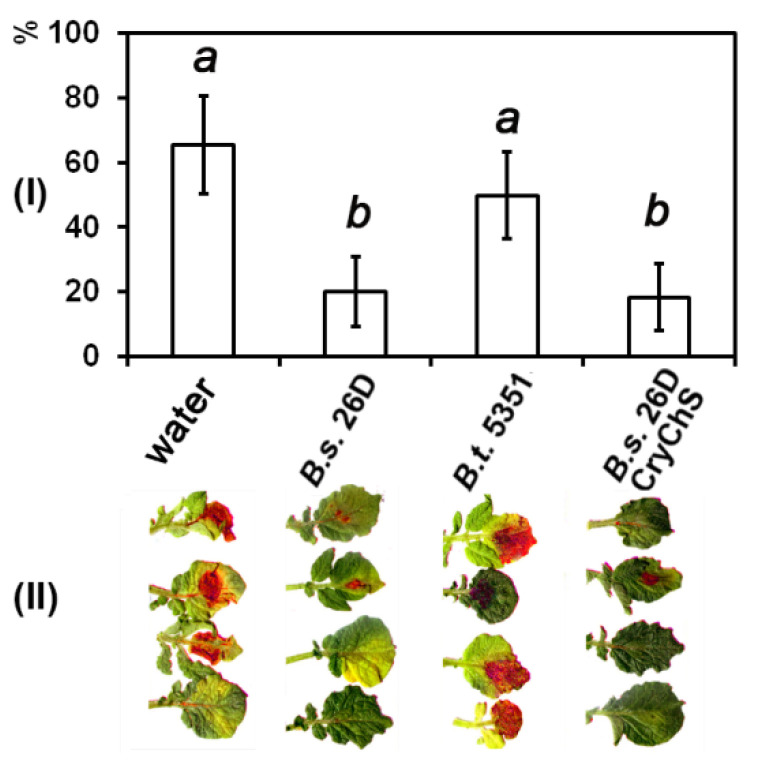
The influence of *Bacillus* strains on late blight disease symptoms on the potato leaves on 10th day after *P. infestans* inoculation. (**I**) Percentage of leaf area with late blight symptoms. Data represented as mean values ± standard error, values followed by the same alphabet are not significantly different from each other according to Student’s test *p* < 0.05. (**II**) Late blight lesions highlighted using ImageJ software (red areas on leaves).

**Figure 2:**
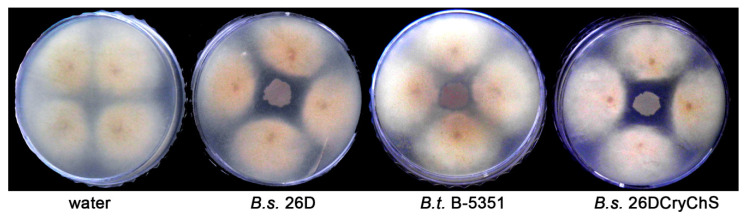
Antagonistic effect of *Bacillus* sp. on pathogenic oomycete *P. infestans* growth in vitro.

The area of late blight-damaged sites on leaves of potato plants containing *B. subtilis* 26D and *B. subtilis* 26DCryChS endophytes reduced in the same manner, whereas the effect of *B. thuringiensis* B-5351 was not substantial (Figure 1). Both *B. subtilis* 26D and *B. subtilis* 26DCryChS strains had antagonistic activity against *P. infestans* and inhibited mycelium growth (Figure 2). The donor of the gene *Btcry1Ia B. thuringiensis* B-5351 did not alter the growth of the oomycete mycelium.

### 2.3. Insecticidal Activity of Bacillus Strains against L. decemlineata Larvae

The *B. subtilis* 26D strain exhibited a moderate insecticidal influence on *L. decemlineata* larvae (18.1%, Table 2), whereas the eating of *B. thuringiensis* B-5351-immersed leaves resulted in about 77% mortality of larvae. Insecticidal activity of the *B. subtilis* 26DCryChS strain was less than the *B. thuringiensis* B-5351 strain when larvae ate surface-contaminated plants (Table 2).

**Figure 3:**
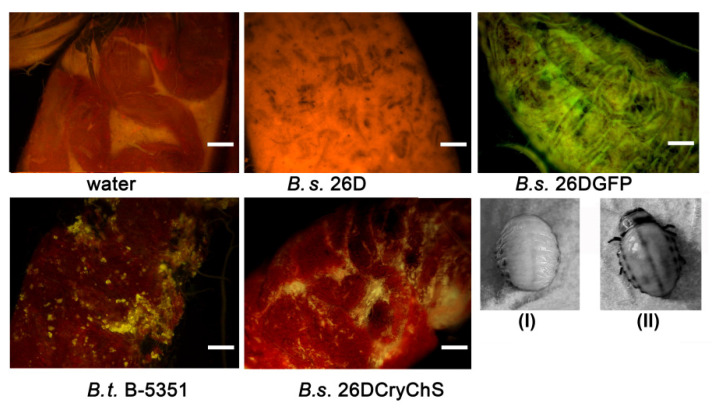
Mesenteron structure of *L. decemlineata* beetles 24 h after feeding of potato plants, which contained cells of *Bacillus* strains. Scale bars, 200 um. (**I**) Healthy larva; (**II**) bacteriosis on alive larva on the 5th day after eating of plants containing *Bacillus* sp.

The strains of *Bacillus* under investigation are able to colonize plant tissues. Consequently, *L. decemlineata* larvae swallowing potato leaves with endophytes in internal tissues become affected both by *Bacillus*-derived insecticides and plant defense mechanisms, primed by endophytes. Thus, feeding of plants containing *B. subtilis* 26D resulted in the death of 38.1% larvae. It is worth noting that the mutualistic relationship between plants and *B. subtilis* 26DCryChS contributed to 80.7% of larval death, and the insecticidal activity of *B. thuringiensis* B-5351 in this case was decreased compared to the case of surface-contaminated leaves. On the fifth day after feeding, larvae showed bacteriosis symptoms (Figure 3II).

Line *B. subtilis* 26DGFP expressing fluorescent protein was obtained to confirm the pathological impact of *B. subtilis* 26D and to visualize its cells in the digestive system of *L. decemlineata*. Beetles were fed with suspensions of the bacteria under investigation (applied to the surface of leaves) to observe the influence of bacterial strains in the same concentration since plants contain different numbers of endophytic cells of these strains. It was found that *B. subtilis* 26D violated the development of the peritrophic membrane and promoted the occurrence of darkened parts on the midgut surface. Fluorescent cells of *B. subtilis* 26DGFP grouped around these sites, as shown in Figure 3. Crystals of Cry-protein were observed in mesenterons of *B. thuringiensis* B-5351- and *B. subtilis* 26DCryChS-fed beetles.

### 2.4. Bacillus Strains Triggered Systemic Resistance in Potato Plants to P. infestans and Beetle L. decemlineata

**Figure 4:**
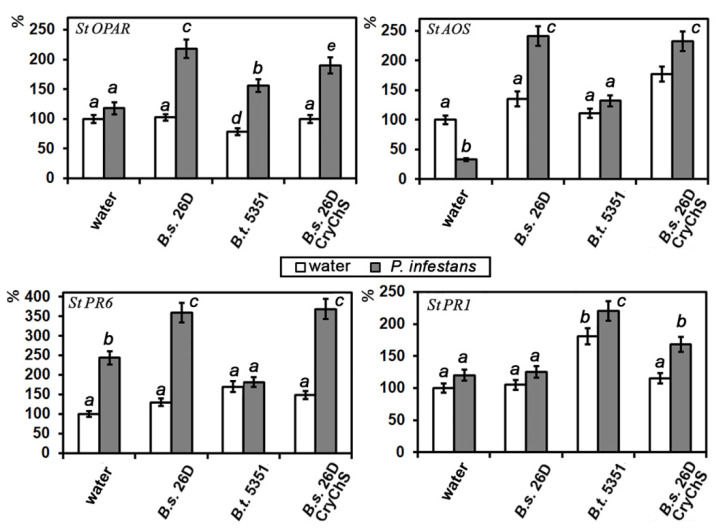
The effect of *Bacillus* strains on transcriptional activity of *StPR6*, *StPR1*, *StAOS*, and *StOPAR* genes in potato plants after 24 h post infection with *P. infestans*. Data presented as mean values ± standard error, values labeled by the same character are not significantly different from each other according to Student’s test *p* < 0.05.

The amount of the transcripts of potato genes *StPR6, StPR1, StAOS,* and *StOPAR*, encoding trypsin inhibitor PR6 (marker of JA-dependent pathway), basic antimicrobial protein PR1 (marker of salicylate-dependent pathway), allene oxide synthase, and 12-12-oxophytodienic acid reductase (enzymes of JA-biosynthesis, sensitive to JA), respectively, in intact and *Bacillus*-containing cells of plants in response to oomycete *P. infestans* or beetle *L. decemlineata* attacks, was investigated. The transcript level of JA-sensitive genes *StPR6*, *StAOS*, and *StOPAR* in non-affected plants containing endophytic *Bacillus* strains in potato plants was not altered (Figure 4), but both *B. subtilis* strains under study decreased the transcript level of *StPR1*. It is important that *B. thuringiensis* B-5351 increased this parameter.

*P. infestans* attack on water-treated potato plants did not lead to significant alterations in *StOPAR* and *StPR1* RNA content and stimulated *StPR6* transcription (Figure 4). All tested *Bacillus* strains positively regulated the transcription activity of JA-sensitive genes *StOPAR*, *StAOS*, and *StPR6* in plants which were infected with the late blight causal agent. Infection of *B. thuringiensis* B-5351-containing plants increased *StOPAR* and *StPR1* transcription but did not influence *StAOS* and *StPR6* genes. It is important that endophytic *B. subtilis* 26DCryChS cells increased levels of both SA- and JA- sensitive genes under study in plants which were attacked by *P. infestans*.

**Figure 5:**
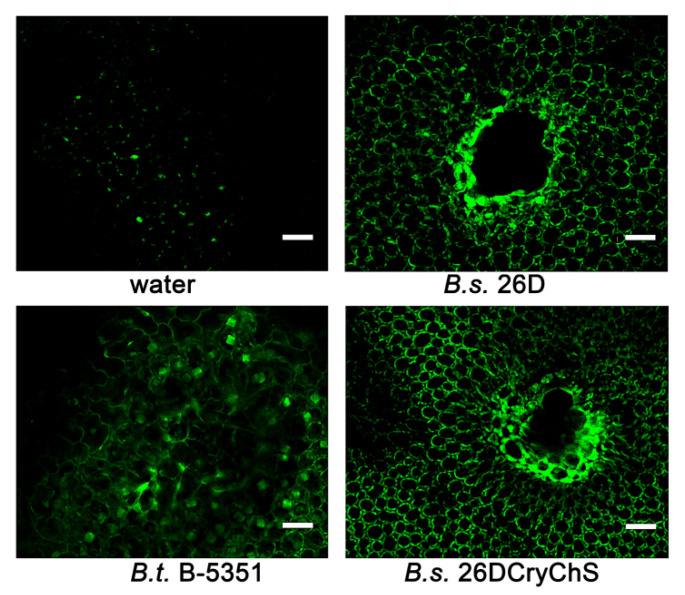
Lignin fluorescence in *P. infestans*-penetrated sites on leaves of water- and *Bacillus* sp.-treated potato plants. Scale bars 20 µm.

It was found that lignin autofluorescence in cell walls around *P. infestans*-penetrated sites on leaves of *B. subtilis* 26D-treated plants was significantly increased relative to water-treated or *B. thuringiensis* B-5351-treated plants (Figure 5). In *B. subtilis* 26DCryChS-treated plants, lignin accumulated in apoplastic spaces and cell walls of the mesophyll and epidermis around infected sites, like in plants under the influence of the original strain.

**Figure 6:**
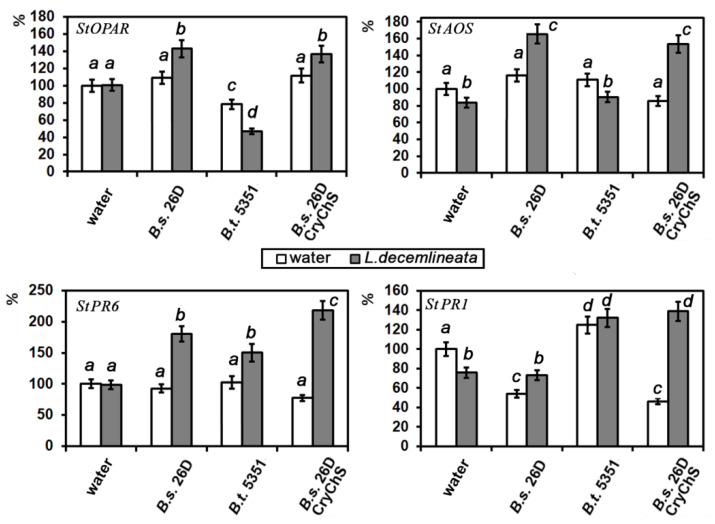
The effect of *Bacillus* strains on transcriptional activity of *StPR6, StPR1, StAOS*, and *StOPAR* genes in potato plants after 24 h post damage caused by larvae of *L. decemlineata*. Data presented as mean values ± standard error, values labeled by the same character are not significantly different from each other according to Student’s test *P* < 0.05.

The transcript level of *StAOS* and *StPR1* genes—unlike *StOPAR* and *StPR6*—was down-regulated by *L. decemlineata* attacks. Treatment of plants with cells of *Bacillus* strains, especially *B. subtilis* 26DCryChS (2-fold), promoted transcription of the *StPR6* gene in infected plants. Presence of endophytic *B. subtilis* 26D strain cells in potato plants enhanced the level of JA-biosynthesis genes and did not have any effect on the RNA content of the *StPR1* gene in plant leaves damaged by larvae of *L. decemlineata* (Figure 6). Conversely, the pest attack on *B. thuringiensis* B-5351-treated plants resulted in the rise of the transcription activity of *StPR1* and did not alter the *StPR6* RNA level. The *B. subtilis* 26DCryChS strain supported the accumulation of both JA- (*StAOS, StOPAR*, and *StPR6*) and SA- (*StPR1*) sensitive genes in plants under the pest attack (Figure 6).

## 3. Discussion

The damage to plants caused by pathogens and insects substantially decreases their productivity and, in some cases, can totally devastate a yield. Approaches based on the use of chemical pesticides have demonstrated enormous gains in controlling populations of some harmful pests, in particular, fungal pathogens and insects. This war is an everlasting competition, since genetic changes in pests often provide restoring their virulence over plant resistance or leave the pest resistant to novel pesticides. The dependence of agriculture on chemical pesticides, the increasing frequency of pest resistance to pesticides, and environmental pollution caused by chemicals are of escalating concern. The search for ways to solve these concerns has resulted in a higher focus on biological control means. Now, it is clear that the development of stable agroecosystems cannot be achieved without the integration of agricultural plants with artificially composed microbiomes or strains, which combine some properties, and which allow to protect plants against combined environmental factors, and, at the same time, will be protected themselves through the development partnerships with the host plants. In consortiums, the set of relationships between microorganisms can lead to incalculable effects in plants under the influence of multiple biotic factors. Thus, we assume that recombinant endophytic *B. subtilis* strains are capable of protecting crops from a number of pathogens or insect pests [8,11,21,26].

Previously, we have shown that the source strain *B. ѕubtilis* 26D effectively protects potato plants against late blight causal agent [9]. The previously obtained *B. subtilis* 26DCгуCһЅ strain, which was developed on the basis of the endophytic *B. subtilis* 26D strain, is able to inhibit the propagation of oomycete *P. infestans* mycelium in vitro and decreased late blight lesion formation on potato leaves as well as the original strain *B. subtilis* 26D (Figure 1 and Figure 2). *B. subtilis* 26D showed proteolytic activity that can cause observed impact of *B. subtilis* 26DCгуCһЅ [13]. Thus, *Bacillus amyloliquefaciens* isolates which produce proteases display antagonistic activity against pathogenic fungi *Fusarium oxysporum, Alternaria alternate*, and *Macrophomina phaseolina* [30]. Chang W.T. et al. [31] showed that *Bacillus cereus* AU004 substantially inhibits the growth of *Pythium ultimum, F. solani*, and *F. oxysporum* mycelium due to the secretion of hydrolytic enzymes.

At the moment, the spore-forming bacterium *B. thuringiensis*, producing insecticidal crystal proteins, is the prevalent commercial agent for insect pest control [18,19]. Sensitivity of the majority of biopesticides based on commercial strains to temperature regime, atmospheric precipitations, and other conditions put bounds on their extensive use. Involvement of endophytic strains in farming techniques can solve the problem [7,18].

As was mentioned above, widespread application of *B. thuringiensis* cells and crystals to manage pest insects has increased the prerequisite for the distribution of pests’, in particular, the Colorado potato beetle, resistance to this entomopathogen [5,6]. Biocontrol agents combining diversified actions on different pest systems can make it possible to reduce the level of pest resistance development. Thus, the great capacity of *Beauveria bassiana* strain GHA and *B. thuringiensis* subsp. *morrisoni* strain *tenebrionis* to act in concert to control *L. decemlineata* was reported (the fast-operating *B. thuringiensis* δ-endotoxin protects defoliation of potato plants and the slow-operating *B. bassiana* increases adult mortality) [32]. The transgenic strain *B. bassiana* expressing *Cyt2Ba* had enhanced virulence against larvae and adults of *Aedes aegypti* and *Ae. albopictus* mosquitoes compared with the wild type of the pathogen. The median lethal time (LT50) for *Ae. aegypti* adults decreased by 33% at the concentration of 10^8^ conidia/mL, 19% at 10^7^ conidia/mL, and 47% at 10^6^ conidia/mL in comparison with the wild type [33].

Besides, it was found previously that *B. subtilis* 26D disarranged *L. decemlineata* larvae microbiome development. Presumably, this effect can be an occasion for the further high mortality rate of the insects [34]. It was found that *B. subtilis* 26D interrupts the integrality of the peritrophic membrane and promotes appearance of dark sites on the midgut surface. Fluorescent *B. subtilis* 26D GFP was placed close by these sites, as shown in Figure 4. Production of Cry1Ia δ-endotoxin has enhanced the insecticidal potential of the *B. subtilis* 26DCryChS strain in comparison to that of the *B. thuringiensis* B-5351 strain. We assumed that it combines the influence of the source strain on the pest microbiome and peritrophic membrane and the influence of the Cry toxin on insect intestines. Probably, the entophytic lifestyle of *B. subtilis* 26DCryChS promotes this effect (Table 2) since the suspension of this strain was less effective than the eating of plants containing endophytes. It is essential to emphasize that the recombinant strain has not got any host specificity, since the increase in the mortality of Russian wheat aphid was observed when insects propagated on wheat seedlings which were grown from *B. subtilis* 26DCryChS strain-inoculated seeds [13].

The suppression of late blight symptoms on potato leaves and the survival rate of larvae of *L. decemlineata* on plants containing cells of *Bacillus* strains under investigation may be associated with the priming of immune reactions in potato plants and ISR [9,21]. The increased mortality of the pest which ate plants containing endophytic *B. subtilis* under investigation compared with ones that ate surface-contaminated leaves testifies to this fact.

Thus, priming is a common characteristic of PGPB-induced plant resistance. Priming initially induces a slight alteration of transcription of defense genes, increasing the plant’s ability to protect itself against further attacks (insect or/and pathogenic pests). In primed plants, subsequent defense responses can be activated more rapidly and effectively [35]. We demonstrated that *Bacillus* under investigation did not increase the number of transcripts of genes encoding PR proteins in potato plants, but *P. infestans* infection or *L. decemlineata* attacks were followed by a great increase in their transcription level (Figure 4 and Figure 6). Observed reactions keep up with the priming effect of microorganisms.

Resistance induced by PGPB is modulated by signal transduction networks in which JA/ET and SA have crucial roles [11,20]. The cross-talk between SA- and JA-sensitive signaling pathways and suppression of JA/ET signaling by SA and/or intermediates of SA-dependent pathways have been reported more than once [21,36]. These interactions permit plants to put forward defense mechanisms which are more effective against certain species with different characteristics of damage, thereby minimizing the development of ineffective and expensive defense mechanisms [20,21], but harmful pests use these arrangements for manipulating plant immunity. Although some non-pathogenic rhizosphere microbes stimulate ISR through the SA-dependent signaling pathway, most beneficial microbes activate ISR through the JA/ET signaling pathway [37]. However, in some cases, ISR can develop through both the SA and JA/ET signaling pathways simultaneously to accord resistance against necrotrophic and hemibiotrophic pathogens [38]. Thus, an increase in SA levels and *AtPR-1* gene expression in plants of *Arabidopsis* after treatment with *B. subtilis* FB17 led to enhanced resistance to *P. syringae* pv. *tomato* [39]. ISR against *Bemisia tabaci* in tobacco induced by *B. subtilis* PY-79 showed a JA-independent mechanism of regulation [37].

Some *B. thuringiensis* strains can prime plants’ defense reactions against pests apart from the insecticidal effect of δ-endotoxin [16]. It was reported that *Bt*-induced resistance to *R. solanacearum* in tomato plants which was dependent on the SA signaling pathway and repressed JA-dependent mechanisms [40]. *B. thuringiensis*-induced plant defense against pathogens and pests is not clear yet [41,42].

Transcriptional activity of the SA- and JA-dependent genes encoding PR proteins was investigated to assess the capability of *Bacillus* strains to manipulate signaling cross-talks in ISR development against *L. decemlineata* and *P. infestans* in potato plants. The *StPR1* gene, which encodes the protein PR1, was reported as the marker of SA-dependent reactions in some plant species [43]. Both *StPR6* and JA-biosynthesis genes were JA-dependent [44]. Previously, it was shown that *B. thuringiensis* and *B. subtilis* strains can induce different transcription patterns in wheat plants [45].

We have previously shown the involvement of JA in potato protective response to the pathogen *P. infestans* [46] and to the pest *L. decemlineata* [47]. The results obtained suggest that the effect of *B. subtilis* 26D was closely linked to the triggering mechanism of ISR caused by JA. Results of Yarullina L.G. et al. [48] showed that inoculation of wheat with *Stagonospora nodorum* increased the level of transcripts of genes encoding the proteinase inhibitor PR6. The rate of the activity of this gene in intact plants of different cultivars correlated with their resistance to the Septoria blotch pathogen. In this work, we found that the source strain and *B. subtilis* 26DCryChS promoted a high level of JA-biosynthesis enzymes in plants under *L. decemlineata* and *P. infestans* attacks. We showed that *B. thuringiensis* B-5351 and *B. subtilis* 26DCryChS strains promoted accumulation of the RNA *StPR1* gene in plants challenged by oomycete *P. infestans* and pest *L. decemlineata* (Figure 4 and Figure 6). Thus, under the influence of the recombinant strain, SA and JA reactions were activated simultaneously. Previously, [38] showed that *B. cereus* AR156 strain treatment led to a higher level of *Arabidopsis* Col-0 plants’ resistance to *Pseudomonas syringae* DC3000 than that in *NahG*, *jar1*, or *etr1* plants. Thus, AR156-mediated ISR is implemented through the simultaneous activation of the JA/ET and SA-sensitive signaling pathways, which made it possible to speak of its cumulative effect on plant immunity in some cases.

It is worth remembering that activation of the SA-dependent *StPR1* gene in plants influenced by *B. thuringiensis* B-5351 was not promoting plant resistance to stresses, but improving it, and then SA- and JA-dependent genes were activated together. Previously, we observed that the simultaneous influence of a low concentration of JA and SA increased potato resistance to *P. infestans* [46] and we assumed that *B. subtilis* 26DCryChS primed plant resistance that engages both pathways.

In our investigation, enhanced lignin deposition around *P. infestans*-penetrated sites on leaves of plants treated with *B. subtilis* strains under investigation was negatively correlated with the dimensions of the lesion areas. Lignin forms a physical barrier on the pathogens and may improve plant resistance against fungal infection [49]. Lignification of plant cell walls and development of a strength barrier to pathogen penetration is one of the most important ISR mechanisms, which could be triggered by PGPB [50]. In [10]’s study, when cucumber roots were treated with a cultural filtrate of hypovirulent *Rhizoctonia* sp., lignin content was increased after pathogen *Colletotrichum orbiculare* penetration in epidermal tissues of cucumber hypocotyls. It was shown that PGPB, in particular, *Bacillus* sp., are capable of stimulating lignin biosynthesis through activation of peroxidases and polyphenol oxidases in infected plants [41,45,51] or pest-damaged plants [11,42]. Genetically engineered plants of maize, expressing the Cry1Ab toxin, showed a higher ratio of lignin accumulation in vascular bundle sheaths and in the sclerenchyma cells near vascular bundles than wild type plants [52].

## 4. Materials and Methods 

### 4.1. Plant, Microbe and Insect Material

**Plants:** sterile *Solanum tuberosum* L. plants (cv. Early Rose). Plants were obtained by microcloning technology and grown in tubes with Murashige and Skoog medium in a KS200 climatic chamber (SPU, Russia) with a 16-h light period (Osram L 36W/77 bulbs, illuminance 8–10,000 lux, Germany) at 20–22 °C for 21 days.

***Bacteria:****B. subtilis* 26D, *B. thuringiensis* B-5351, and previously obtained *B. subtilis* 26DCryChS which contained the *Btcry1Ia* gene encoding Cry1Ia δ-endotoxin from *B. thuringiensis* B-5351 [13] strains held in the collection of the Laboratory of Biochemistry of Plant Immunity of the Institute of Biochemistry and Genetics UFRC [53] were used. Bacteria were grown on Luria-Bertani (LB) medium (1% tryptone, 0.5% yeast extract, 0.5% NaCl) at 20–22 °C.

***System “Plant+endophyte”*:** 7-days-old plants were inoculated with 5 μL of *Bacillus* strains cell suspension on the stem zone adjacent to the zone of adventitious roots formation as it was described previously [34]. Concentration of bacterial cells was 10^8^ cells/mL. Plants were grown in a gnotobiotic system for 14 days.

***L. decemlineata***: third instar larvae and adults were taken from seed potato (Birsk experimental farm, the Republic of Baskortostan, the Birsk region, South Ural, 55°25′52.6′′ N 55°35′58.3′′ E).

***P. infestans****:* MO-8 (race 1.2) culture kindly provided by U.T. Diakov from the collection of the Biological Faculty, Moscow State University and stored in the collection of our laboratory was used.

### 4.2. Evaluation of Endophytic Properties of Bacillus Strains

On the 7th day after planting, sterile potato plants were inoculated with the suspension of *Bacillus* sp. cells (“Plant+endophyte”). After 14 days of co-culturing, leaves and stems of plants were immersed for 3 min in 70% EtOH; after this, for 3 min in a 0.03% solution of hydrogen peroxide, and then the samples were washed with sterile water three times. Sterilized potato leaves and stems (200 mg fresh weight) were homogenized in sterile bags using a BagMixer 400 W blender (Interscience, Saint Nom, France) with 2 mL sterile water added. Two consecutive 10-flast dilutions of the resultant homogenate were then performed. Aliquots (100 μL) were spread over the surface of potato-glucose agar with an L-shaped spatula until complete drying. After 24 h, Petri dishes were analyzed for the count of CFU. The ability of strains under investigation to colonize the internal plant tissues was expressed in CFU per gram of wet weight. In order to verify and confirm the identity of bacterium isolates, obtained from surface-sterilized leaves, and original strains, pure cultures were analyzed by RAPD-PCR.

### 4.3. Evaluation of the Ability of Bacillus Strains to Defend Potato Plants against P. infestans

Leaves of 21-day potato plants which were previously treated with bacteria (“Plant+endophyte”) or water were placed in Petri dishes on 0.04% benzimidazole-soaked cotton wool. Potato leaves were infected by spraying with 5 μL of *P. infestans* spore suspension (10^5^ spores/mL). The development of symptoms on potato leaves was evaluated on the seventh day after infection by measurement of the lesion area on leaves and fixing images with the SP-800UZ Image camera (Olympus, Bekasi, Indonesia). Late blight signs became visible 1 day after the infection, and their extent was estimated by percent of affected area of the leaf blade on the 10th day after inoculation. To measure the area, leaves were photographed, and the images analyzed using the ImageJ program (NIH, USA) [30]. Ten plants of each variant group were selected for analysis, and three leaves from one plant were collected at the indicated time point per biological replicate.

Whole plants were infected by spraying with 25 5 μL of *P. infestans* spore suspension (10^5^ spores/mL) for the investigation of local and systemic immune reactions.

To evaluate the antifungal activity of the strains, 5 mm-across slices of *P. infestans* mycelium were placed on 4 sides of Petri dishes with potato-glucose agar. Bacterial strains under investigation were placed individually in the center of the medium. Plates were cultured at 28 °C for 7 days. The test was performed in triplicate.

### 4.4. Evaluation of Bacillus Strains Insecticidal Activity against L. decemlineata 

Then, the leaves of the control and endophytes-containing plants with petioles covered by paper gauze and inserted into tubes with water were placed in glass vessels and beetles were fed on potato leaves of these plants (1 beetle on 1 plant). Potato plants with surfaces contaminated by *Bacillus* strains were used to investigate the direct insecticidal effect of strains. Sterile potato plants were dipped into bacterial suspensions (10^8^ cells/mL) for 1 min and were exposed to beetle attack at once as mentioned above.

Some adults were selected for gut investigations (Biozero BZ-X700 (“Keyence”, Japan)). Subsequently, beetles in all vessels ate non-infested plants to remove *B. subtilis* 26D-containing intestinal contents. The control groups of both variants were fed with potato leaves of plants treated with sterile water. Percentage of dead beetles was assessed. 

### 4.5. Construction of Fluorescent B. subtilis 26DGFP

**Figure 7:**
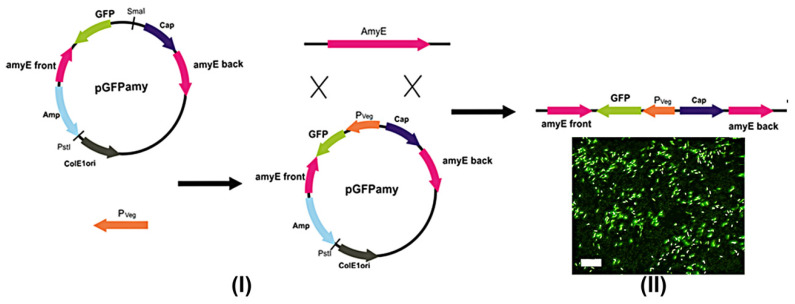
(**I**) Construction of the gene engineering plasmid pGFPamyPveg for *B. subtilis* 26D transformation. (**II**) *B. subtilis* 26DGFP fluorescens (Scale bar 50 nm). AmyE’ front, and amyE back—5’ and 3’ parts of *B. subtilis* 168 a-amylase gen; Cap encodes chloramphenicol acetyl transferase; Amp encodes beta-lactamase; SmaI and PstI—restriction sites, ColE1ori—origin of replication in *E. coli*.

Genetically engineered construction on the basis of the pGFPAmy plasmid which contained a gene marking the green fluorescent protein GFPmut3 (excitation/emission-482/502 nm) was used for *B. subtilis* 26D recombination. Promotor Pveg of *B. subtilis* was amplificated by Pfu-polymerase using primers PvegF 5′-GGAGTTCTGAGAATTGGTATGCCTTAT-3′ and PvegR 5′- ACTACATTTATTGTACAACACGAGCCC-3′. pGFPamy was treated with endonuclease SmaI and ligated with Pveg [54]. Plasmid PGFPamyPveg was transformed into recipient strain *B. subtilis* 26D, giving ectopic insertion at the AmyE locus by a double cross-over recombination event (Figure 7I). Bacteria were selected on LB medium containing 100 µg/mL chloramphenicol. Fluorescence was observed using the microscope Biozero BZ-X700 (“Keyence”, Japan) with the standard filter (Figure 7II).

### 4.6. RNA Isolation and the Reverse Transcription Quantitative Polymerase Chain Reaction (qPCR) 

Total RNA was isolated from plants+endophyte and sterile plants 24 h post-infection/pest impact with the TRI^®^ reagent (Sigma-Aldrich, St. Louis, MO, USA). RNA concentration was measured using the Smart Spec Plus spectrophotometer (BioRad, Hercules, CA, USA). Concentrations of RNA in samples were equalized. The first cDNA strand was synthesized using oligonucleotide primers and M-MLV reverse transcriptase (Thermo Scientific, Madrid, Spain).

The obtained cDNA was diluted 5-fold and used for quantitative PCR (qPCR). Quantitative PCR was performed by polymerase chain reaction in real time using a set of predefined reagents N’,N’-dimethyl-N-(4-((E)-(3-methyl-1,3-benzothiazol-2-ylidene)methyl)-1-phenylquinolin-1-ium-2-yl)-N-propylpropane-1,3-diamine (SYBR Green I) (Synthol, Russia) and the device CFX Connect Real-Time PCR Detection System (Bio-Rad, Hercules, CA, USA). qPCR was run according to the following program: 50 °C for 2 min; 95 °C for 10 min; 40 cycles of 95 °C for 15 s, and at 60 °C for 1 min. After the final PCR cycle, a melting curve analysis was conducted to determine the specificity of the reaction (at 95 °C for 15 s, 60 °C for 1 min, and 95 °C for 15 s). The efficiency of primer pairs was evaluated using 10-fold cDNA dilution series. The expression of each target gene is presented as fold change normalized to the reference gene *StAct* (Potato Actin) and relative to the untreated control sample. iCycler iQ5 Real-Time Detection System software (BioRad, USA) was used for data analysis. The primers used are given in Table 3.

### 4.7. Lignin Autofluorescence Registration

Localizations of lignin in leaves were studied 24 h after plant inoculation. Leaves were fixed with 96% ethanol at 4 °C for 4 h, and were placed in the mixture of ethanol/glycerin 1:1. Lignin autofluorescence on infected leaves was investigated with the laser scanning confocal microscope LSM-510 based on the inverted microscope Axiovert 200 M (“Carl Zeiss”, Oberkochen, Germany). For excitation of autofluorescence, an argon laser of 30 mW with a wavelength of 488 nm, dichroic 490 nm mirror, and 505 nm transmission filter was used [55]. 

### 4.8. Statistics

At least three biological replications in three technical repetitions each were examined in all. At least three biological replications in three technical repetitions each were examined in all experiments, including experiments with cDNA. Data presented are mean values with standard errors (±SE). Means were compared using analysis of variance (ANOVA) with *p* ≤ 0.05. Different letters on figures’ labels mean significant differences between treatments according to Student’s test at *P* < 0.05. The program Statistica 12.0 (Stat Soft, Moscow, Russia) was used.

## 5. Conclusions

Production of insectotoxins in recombinant lines which were obtained using endophytic bacterial strains seems to be one of the most reasonable techniques to create improved ecologically friendly biocontrol agents for pest management [17]. Artificial regulation of plant defense responses to herbivorous insects and pathogens, which frequently attack them simultaneously under field conditions, stands in need of continuous investigations. PGPB, including endophytic strains, could regulate SA- and JA-sensitive defense mechanisms in compliance with the type of damage or pathogen/pest attacks means [42,45]. Thus, the strain *B. subtilis* 26DCryChS is capable of stimulating both SA- and JA-dependent mechanisms in potato plants while host plants are under pest or pathogen attack.

Production of Cry1Ia δ-endotoxin in the endophytic source strain led to the development of a strain with integrated fungicidal (Figure 3) and insecticidal (Table 2) activities and capability to prime potato plants’ defense reactions through involvement of both SA- and JA-dependent pathways (Figure 4 and Figure 6). Endophytic *B. subtilis* 26DCryChS showed improved protective efficacy on potato plants against both oomycete *P. infestans* and *L. decemlineata.* The use of biocontrol agents based on *B. subtilis* 26DCryChS could be an alternative strategy of integrated plant protection.

## Figures and Tables

**Table 1:** Content of *Bacillus* sp. in plant tissues after surface sterilization, CFU*10^3^/g of plant wet mass (14th day post inoculation).

Variant	CFU*10^3^/g
water (0)	-
*B. subtilis* 26D	420 ± 63a
*B. thuringiensis* B-5351	7.5 ± 0.25b
*B. subtilis* 26DCryChS	487 ± 53a

Note: Data presented as mean values ± standard error, values labeled by the same character are not significantly different from each other according to Student’s test *p* < 0.05.

**Table 2:** Effect of feeding on surface contaminated with *Bacillus* spp. or containing endophytic *Bacillus* sp. cells in the internal tissues of potato plants on *L. decemlineata* larvae mortality.

Variant/Parameter	Mortality, % (7th Day after Feeding)	
	Surface-Contaminated Plants	Plant + Endophyte
Water	4.1 ± 0.7a	6.7 ± 0.5a
*B. subtilis* 26D	18.1 ± 3.5b	38.1 ± 5.2b
*B. thuringiensis* B-5351	76.7 ± 6.35c	55.6 ± 5.8c
*B. subtilis* 26DCryChS	48 ± 11.8d	80.7 ± 13.3d

Note: Data presented as mean values ± standard error, values labeled by the same character are not significantly different from each other according to Student’s test *P* < 0.05.

**Table 3:** Primers used for quantitative PCR.

Gene	Gene Product	NCBI Access Number	Primers
*StPR6*	Trypsin inhybitor, PR-6	AY089962	F 5’-gctgaggattggtgagaggta-3’ R 5’-ccacatcaccataatccaact-3’
*StPR1*	basic antimicrobial protein PR-1	AY050221	F 5’-tgggtggtggttcatttcttgt-3’ 5’-catttaattccttacacatcataa-g-3’
*StAOS*	allene oxide synthase	DQ174273	F 5’-gcacactttccctctaccttac-3’ R 5’-ccaagtttctccgcttcatcta-3’
*StOPAR*	12-12-oxophytodienic acid reductase	JN241968	F 5’-gggatacacagattaccctttcc-3’ R 5’- tcgggcttcacaagttcttac-3’
*StAct*	actin	X55749	F 5’-gatggtgtcagccacac-3’ R 5’-attccagcagcttccattcc-3’

## Abbreviations

CFU	colony-forming units
ET	ethylene
JA	jasmonic acid
PGPB	plant growth-promoting bacteria
PR proteins	pathogens-related proteins
SA	salicylic acid
